# Centrosome amplification promotes cell invasion via cell–cell contact disruption and Rap-1 activation

**DOI:** 10.1242/jcs.261150

**Published:** 2023-11-01

**Authors:** Anu Prakash, Shishir Paunikar, Mark Webber, Emma McDermott, Sri H. Vellanki, Kerry Thompson, Peter Dockery, Hanne Jahns, James A. L. Brown, Ann M. Hopkins, Emer Bourke

**Affiliations:** ^1^ Lambe Institute for Translational Research, Discipline of Pathology, Centre for Chromosome Biology, University of Galway, Galway H91 V4AY, Ireland; ^2^ Centre for Microscopy and Imaging, Discipline of Anatomy, School of Medicine, University of Galway, Galway H91 W5P7, Ireland; ^3^ Pathobiology Section, School of Veterinary Medicine, University College Dublin, Dublin, Ireland; ^4^ Department of Biological Sciences, University of Limerick, Limerick V94T9PX, Ireland; ^5^ Limerick Digital Cancer Research Centre (LDCRC) and Health Research Institute, University of Limerick, Limerick V94T9PX, Ireland; ^6^ Department of Surgery, Beaumont Hospital, Royal College of Surgeons in Ireland, Dublin D09 DK19, Ireland

**Keywords:** Centrosome amplification, Rap-1, Invasion, Metastases, CAM assay, Breast cancer

## Abstract

Centrosome amplification (CA) is a prominent feature of human cancers linked to tumorigenesis *in vivo*. Here, we report mechanistic contributions of CA induction alone to tumour architecture and extracellular matrix (ECM) remodelling. CA induction in non-tumorigenic breast cells MCF10A causes cell migration and invasion, with underlying disruption of epithelial cell–cell junction integrity and dysregulation of expression and subcellular localisation of cell junction proteins. CA also elevates expression of integrin β-3, its binding partner fibronectin-1 and matrix metalloproteinase enzymes, promoting cell–ECM attachment, ECM degradation, and a migratory and invasive cell phenotype. Using a chicken embryo xenograft model for *in vivo* validation, we show that CA-induced (+CA) MCF10A cells invade into the chick mesodermal layer, with inflammatory cell infiltration and marked focal reactions between chorioallantoic membrane and cell graft. We also demonstrate a key role of small GTPase Rap-1 signalling through inhibition using GGTI-298, which blocked various CA-induced effects. These insights reveal that in normal cells, CA induction alone (without additional oncogenic alterations) is sufficient to confer early pro-tumorigenic changes within days, acting through Rap-1-dependent signalling to alter cell–cell contacts and ECM disruption.

## Teaser

**Summary:** Centrosome amplification is sufficient, without additional pro-oncogenic alterations, to drive early tumorigenic change in a normal breast epithelial background.

## INTRODUCTION

Centrosomes play essential roles in averting genomic instability by regulating cell cycle control, spindle assembly and ultimately cell replication. Centrosome amplification (CA), a numerical centrosomal defect (three or more centrosomes per cell) is a hallmark feature of many high-grade human tumours (Chan, 2011). CA is triggered by well-characterised mechanisms, including cytokinesis failure, dysregulation of centrosome duplication proteins and pericentriolar material (PCM) fragmentation (reviewed in Godinho et al., 2014). Additionally, extra centrosomes are directly induced *in vitro* by DNA damage and defects in DNA repair pathways linking CA to inherent genetic instability of tumour cells (Bourke et al., 2010, 2007; Dodson et al., 2004). However, it is increasingly evident that aberrant centrosome numbers are not necessarily a downstream consequence of tumorigenesis and centrosome abnormalities directly perpetuate genomic instability by promoting chromosome missegregation (Cosenza and Kramer, 2016; Ganem et al., 2009) and cellular invasion via cell-autonomous and non-autonomous mechanisms (Adams et al., 2021; Arnandis et al., 2018). CA induction promotes tumorigenesis *in vivo* (Coelho et al., 2015; Levine et al., 2017; Sercin et al., 2016) and has been observed in pre-malignant metaplasia, with CA rates increasing concurrently with disease progression to dysplasia, and is often accompanied by p53 mutation and loss (Lopes et al., 2018; Pihan et al., 2003; Segat et al., 2010). Together, this evidence suggests that CA plays a crucial role in early tumour initiation and malignant progression.

In its capacity as the major microtubule-organising centre (MTOC), the centrosome orchestrates microtubule (MT) assembly pending mitosis while also controlling cell positioning, polarity and motility through its links to the acto-myosin cytoskeleton, cell–cell and cell–extracellular matrix (ECM) junctions (Bettencourt-Dias, 2013; Bornens, 2012). Cell–cell attachments are key to maintaining barrier function in polarised epithelia, and are mediated by highly specialised junctional complexes consisting of tight junctions, adherens junctions, desmosomes and gap junctions (Garcia et al., 2018). Crucially, the formation and regulation of these junctions is dependent on their interactions with cytoskeletal polymers of MTs and actin, pools of which can nucleate from the centrosome (Farina et al., 2016, 2019; Vasileva and Citi, 2018). Therefore, we hypothesise that abnormalities of the centrosome, structural or numerical, might have the potential to impact on cell–cell junction function. Indeed, recent studies have shown that structural centrosome anomalies induced by ninein-like protein (NLP) overexpression in MCF10A breast epithelia, disrupt epithelial polarity and cell–cell contacts via dysregulation of E-cadherin localisation within adherens junctions mediated by small GTPase Rac1 and Arp2/3 signalling (Ganier et al., 2018). This leads to increased cell stiffness, detachment from the epithelium and cell budding towards the ECM. CA induced by PLK4 overexpression in MCF10A cells generates increased MT nucleation and polymerisation, also causing Rac1 activation and Arp2/3-dependent actin polymerisation, leading to invasive lamellipodia formation in 3D cultures. The invasive phenotype is accompanied by loss of cell–cell adhesion, disruption of junction size and positioning as well as dysregulation of E-cadherin junctional localisation (Godinho et al., 2014). In human umbilical vein endothelial cells (HUVECs), CA induction alters cell polarity and perturbs the luminogenesis of newly formed endothelial sprouts accompanied by chaotic localisation of vascular endothelial (VE)-cadherin, resulting in the disruption of adherens junction stability (Buglak et al., 2020). Although data suggests the CA-induced metastatic phenotype is mediated by perturbations of cell–cell and cell–ECM contacts, clear evidence of the mechanism remains to be identified and validated.

Another central step towards local invasion of tumour cells into adjacent tissue is the degradation of ECM components via matrix metalloproteinase (MMP) secretion (Kessenbrock et al., 2010). This family of zinc-dependent endopeptidases are involved in physiological tissue remodelling as well as pathological ECM breakdown within the tumour microenvironment. Emerging evidence of the role of CA in cell migration and invasion points to a possible link between CA and increased MMP expression. *In vivo*, tumours derived from the injection of p53-null tetraploid cells into mice are associated with high levels of centrosome amplification, accompanied by amplification of an MMP gene cluster (Fujiwara et al., 2005). Furthermore, treatment with the broad-spectrum MMP inhibitor marimastat (BB-2516) decreased the fraction of CA-induced invasive acini in MCF10A 3D cultures (Godinho et al., 2014). Proposed intracellular mediators of CA-induced effects include the subfamilies of small GTPases, Rho and Ras, which play roles as molecular switches integrating signals from the membrane to actin cytoskeletal changes to a wide range of effector proteins of migration and invasion, including the expression of MMPs (Sahai et al., 2001). It has been shown that the Rho-GTPase Rac1 is activated by CA induction in breast epithelial cells and that a Rac1 inhibitor (NSC23766) partially rescued the invasive CA-induced phenotype (Godinho et al., 2014). However, the complex and multifaceted range of signalling pathways activated by CA-induction, which facilitates cellular invasion and ECM remodelling, requires further detailed characterisation.

In this study, we investigate how CA induction alone can drive the initial stages of tumorigenesis by promoting cell invasiveness (in both non-tumorigenic and tumorigenic breast epithelial cells) via alteration of extracellular contacts (cell–cell and cell–ECM) and dysregulated MMP and TIMP production, mediated through Rap1 signalling. The results were validated using both an *in vitro* semi-3D long-term cell culture system and an *in vivo* (chick embryo xenograft) model. *In vivo* implantation of CA-induced (+CA) MCF10A cells resulted in cell invasion into the chorioallantoic membrane and a vigorous proliferative focal reaction involving immune cell infiltration.

## RESULTS

### CA increases cellular migration and invasion *in vitro* and in an *in ovo* chick embryo xenograft model

**Fig. 1.**
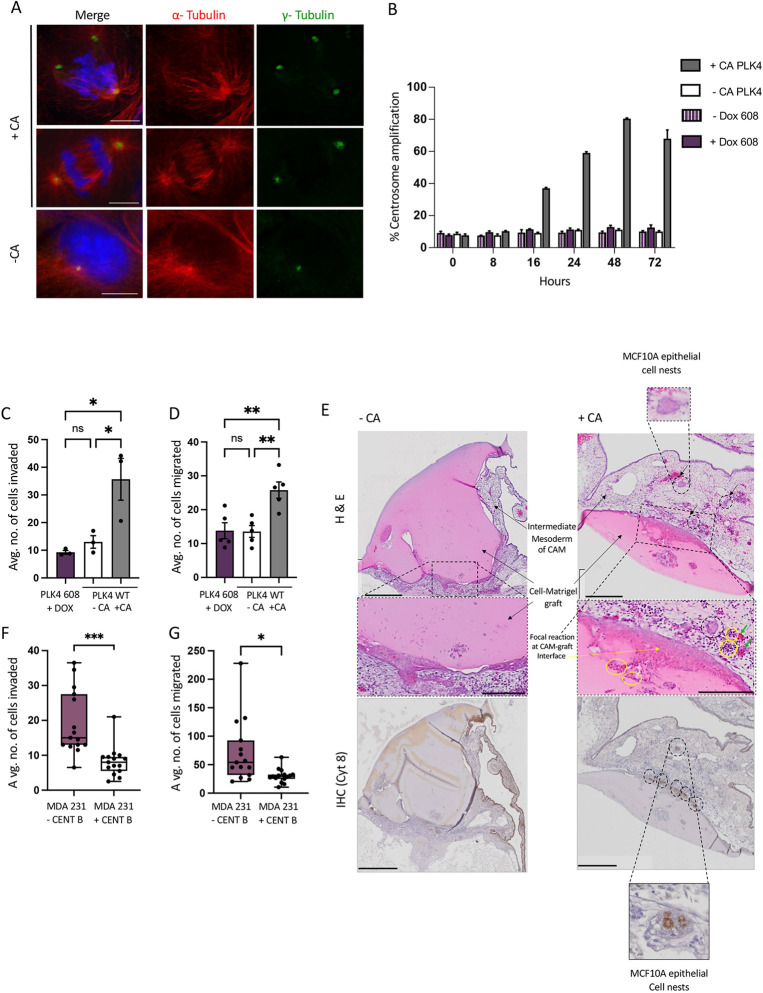
**CA increases *in vitro* and *in vivo* migration and invasion, and blocking CA diminishes the metastatic characteristics of MDA-MB-231 cells.** (A) Representative images of immunofluorescently stained −CA and +CA MCF10A cells fixed with methanol/5 mM EGTA solution and stained for MTs (α-tubulin, red), centrosomes (γ-tubulin, green) and counterstained with DAPI (DNA, blue). The +CA panels show a multipolar mitotic (mitotic catastrophe) orchestrated by amplified centrosomes and clustered centrosomes in a pseudobipolar mitotic. The −CA panel shows a cell with a normal centrosome complement of 2. Scale bar: 10 μm. Images are representative of *n*=3 independent experiments. (B) Fluorescence microscopy quantification of the percentage of +CA cells at each time point post-Dox treatment; normal centrosome number (≤2 per cell) and amplified centrosomes or CA (≥3 per cell). Bar graphs represent mean±s.e.m. from three independent experiments; ≥200 cells/time point. (C,D) Transwell invasion (C) and migration (D) assay results showing that CA induction significantly increases cellular invasion and migration in MCF10A PLK4 cells compared to controls −CA PLK4 and DOX+ PLK4^1-608^. The bar graph represents the mean±s.e.m. number of cells invaded and migrated from four independent experiments. *P≤0.05; **P≤0.01; ns, not significant (one-way ANOVA and Bonferroni's multiple comparison test). (E) Representative images from four independent experiments for the chicken xenograft assay for the MCF10A −CA and +CA cell–Matrigel graft. Images show H&E staining (upper panels; Matrigel staining is pink) and immunohistochemical staining for cytokeratin 8 (Cy8; lower panels) on formalin-fixed paraffin-embedded tissue sections −CA and +CA MCF10A PLK4 cells–Matrigel grafts. The +CA graft shows moderate to strong chicken-graft focal reaction (yellow arrow), with multifocal heterophil infiltration in the intermediate mesoderm layer (yellow circles), invasion of epithelial cell nests into chicken mesodermal layer (black arrows and circles) and dilated lymphatics in the intermediate mesoderm layer (green arrows). Mild to moderate focal reaction of the CAM to the graft was noted in −CA grafts. The corresponding IHC (lower panels) shows that the invaded epithelial cell nests were positive for Cy8 (dotted circles). Scale bars: 500 µm (top and bottom panels); 250 µm (middle panels). (F,G) Transwell invasion (F) and migration assays (G) show that blocking endogenous CA with 150 nM Centrinone B for 16 h in the TNBC cell line MDA-MB-23 significantly decreased cellular invasion and migration. Migrated or invaded cells were quantified with DAPI staining and the cells counted using immunofluorescence images from five different fields at 20× magnification. The box represents the 25–75th percentiles, and the median is indicated. The whiskers show the data spread from two independent biological repeats. *P≤0.05, ***P<0.001 (unpaired two-tailed Student's *t*-test). WT, wild-type.

Polo-like kinase (PLK4) is the master regulator kinase of the centrosome duplication cycle (Bettencourt-Dias et al., 2005; Habedanck et al., 2005). Non-tumorigenic breast epithelial MCF10A PLK4 cell lines employed in the current study were engineered with doxycycline (Dox)-inducible PLK4 transgenes. Previous studies using these cell lines have shown that Dox-induced wild-type PLK4 expression induces CA (+CA MCF10A) whereas a Dox-induced truncated PLK4 variant PLK4^1-608^ (lacking the C-terminal centrosome localisation domain; amino acids 1–608) does not induce CA (Godinho et al., 2014). Here, Dox treatment of MCF10A PLK4 cells (+CA MCF10A) led to a significant (*P*≤0.02) CA induction in 80% (±1.26 s.e.m.) of cells after 48 h (Fig. 1A,B). We measured cell invasion and migration post-CA induction using Matrigel-coated and uncoated Transwell assays, respectively. +CA MCF10A PLK4 cells showed a significant (*P*≤0.05) ∼3-fold increase in invasion (Fig. 1C), and a significant (*P*≤0.02) ∼2-fold increase in migration (Fig. 1D) compared to both untreated controls and PLK4^1-608^. To independently validate that these findings are dependent on CA induction, we induced CA in wild-type MCF10A by CDK1 inhibition, as previously demonstrated (Bourke et al., 2010) using the CDK1 pharmacological inhibitor RO3306 (Steere et al., 2011). Confirming previous reports, RO3306 treatment significantly induced CA (*P*≤0.02), migration and invasion in a MCF10A wild-type cell line (*P*≤0.05) (Fig. S1A–C).

**Fig. 2.**
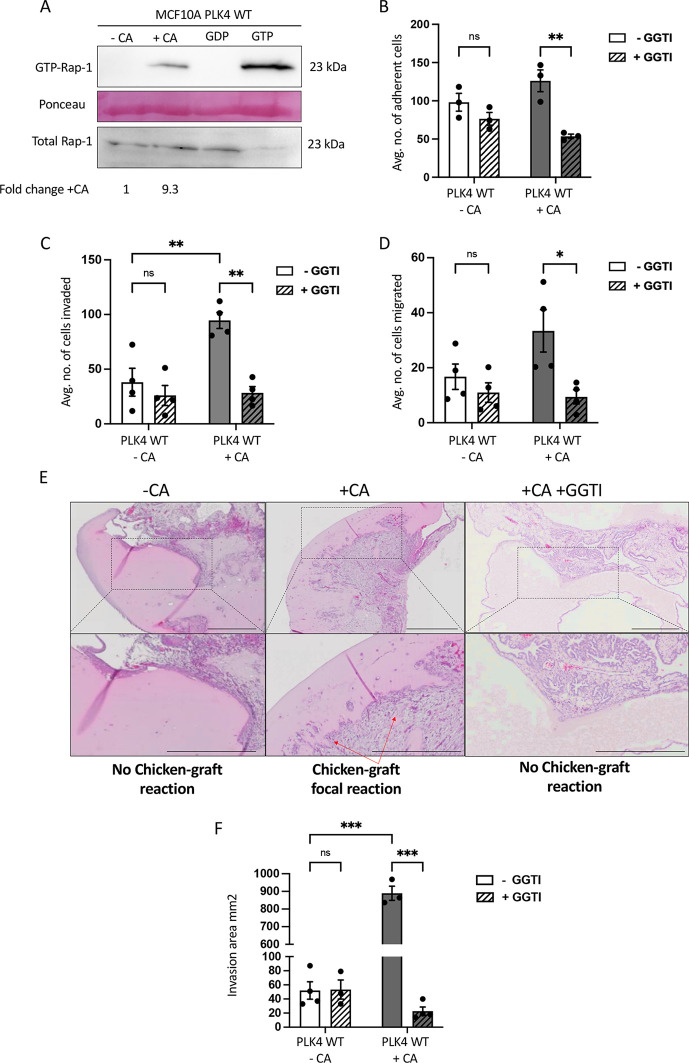
**CA-induced migration, invasion and cell–ECM attachment involves activation of Rap1.** (A) Western blot of Rap1 pulldown assay in MCF10A PLK4 showing CA induced increased GTP-bound Rap1. Negative (GDP) and positive (GTPγS) controls on untreated cell lysates were included. Ponseau stain is included as a loading control for the pulldown. Total Rap1 in 60 µg of total cell lysates is shown in the bottom panel. The image shown is representative of *n*=3 independent repeats and the fold-change represents the quantification of +CA and −CA bands by densitometry. (B) CA increases MCF10A cell–ECM adherence in a Rap1-dependent manner. Cell adhesion assays was performed on MCF10A PLK4 cells 48 h post-Dox (2 μg/ml) or without Dox, and 3 h treatment with or without 10 µM GGTI-298. Cells bound to the matrix were quantified with Crystal Violet staining; five 20× fields per well were counted and the bar graph represents mean±s.e.m. from four independent experiments. **P≤0.01; ns, not significant (two-way ANOVA and Bonferroni's multiple comparison test). (C,D) Rap1 inhibition significantly decreased CA-induced cellular invasion and migration. The bar graphs represent mean±s.e.m. number of cells invaded and migrated, quantified by Crystal Violet staining in five random 20× fields per insert from four independent experiments. *P≤0.05; **P≤0.01; ns, not significant (two-way ANOVA and Bonferroni's multiple comparison test). (E) Representative H&E images of the chicken xenograft assay with cell–Matrigel graft. MCF10A PLK4 cells were xenografted 48 h post-Dox (2 μg/ml) or without Dox treatment and 3 h treatment with or without 10 µM GGTI-298. Rap1 inhibition decreased the CA-induced bidirectional chicken-graft focal reaction *in vivo*. Scale bars: 500 µm. (F) Area (mm^2^) of chicken-graft focal reaction was quantified in scans of MCF10A grafts in +CA or –CA with or without GGTI-298 using Olympus OlyVIA software. Bar graphs represent mean±s.e.m. from three independent experiments. ***P≤0.001; ns, not significant (two-way ANOVA and Bonferroni's multiple comparison test). WT, wild-type.

To investigate how CA induction could initiate early tumorigenic change *in vivo*, we employed a chicken embryo xenograft model. The chorioallantoic membrane assay (CAM assay), is a rapid, cost effective *in vivo* model for the study of tumorigenesis, angiogenesis, invasion and metastases (reviewed in DeBord et al., 2018; Komatsu et al., 2019; Miebach et al., 2022; Ribatti, 2014). The CAM assay has been used to study angiogenesis and tumour cell invasion in breast, pancreatic, colorectal, prostate, brain and ovarian cancers using both human tumour cell line-derived xenografts as well as patient-derived xenografts (Aaltonen et al., 2022; Harper et al., 2021; Lokman et al., 2012). The highly vascularised chicken chorioallantoic membrane of the fertilised chicken egg (onto which the MCF10A cells are grafted) consists of the chorionic epithelium (the outer layer) with a superficial vascular plexus, and the intermediate mesoderm layer and the allantoic epithelium (inner layer). The CAM assay allows characterisation of extremely early changes post-CA induction, not previously observable using *in vivo* mouse CA models (Coelho et al., 2015; Levine et al., 2017; Sercin et al., 2016). Matrigel-embedded control or +CA MCF10A cells were implanted onto the chick chorioallantoic membrane and allowed to grow for 5 days. Histological evaluation showed that the +CA MCF10A PLK4 invaded into the chorionic epithelium (CE) (at the cell–Matrigel interface) (black arrows, Fig. 1E) inducing a multifocal reaction (ranging from moderate to marked) of the CE including proliferation of mesodermal cells, which spread extensively into graft area (red arrows, Fig. 1E), multifocal heterophil infiltration (red circled area, Fig. 1E), and dilated lymphatics in the intermediate mesoderm layer (green arrows, Fig. 1E). Additional representative images of Matrigel–CE reactions are shown in Fig. S2A–C. +CA MCF10A cells (labelled with anti-human cytokeratin 8) invaded into the chicken tissue, forming epithelial cell nests within the intermediate chicken mesodermal layer (Fig. 1E, dotted circles and enlarged panel). Quantification of CAM invasion into the Matrigel–cell graft showed that CA increased the area of invasion significantly compared to −CA grafts (*P*≤0.001, see Fig. 2F). Interestingly, histological analysis indicated that +CA MCF10A sections displayed clear secondary tumour characteristics including haemorrhaging, necrosis and calcification at the CAM–Matrigel interface (Fig. S3A–C). Taken together, these results show that CA induction alone in non-tumorigenic cells is sufficient to induce a migratory and invasive cellular phenotype (both *in vitro* and *in vivo*), and induction of secondary tumour-associated changes in the surrounding tissue *in vivo*.

To evaluate how CA contributes to an invasive phenotype, we utilised metastatic MDA-MB-231 cells (triple-negative breast cancer; TNBC), which have endogenously high CA levels (Choe et al., 2018). CA was blocked using the PLK4-specific inhibitor Centrinone B. At <48 h Centrinone B treatment has been previously reported to reduce centrosome and centriole numbers, reverting CA cell lines to a normal centrosome complement (one or two centrosomes), with >48 h treatment leading to complete centrosome depletion (Denu et al., 2020, 2018; Marteil et al., 2018; Wong et al., 2015). Centrinone B-treated MDA-MB-231 had CA reduced significantly (*P*≤0.05) from 39.75% (±4.6) to 10.25% (±0.35) (mean±s.e.m., *n*=2; data not shown). Furthermore, Centrinone B-treated MDA-MB-231 cells showed significantly (*P*≤0.001) reduced invasion (2.5-fold), and significantly (*P*≤0.05) reduced migration (2.7-fold) (Fig. 1F,G).

To further explore the observed Centrinone B effects on migration or invasion, a second invasive TNBC cell line, MDA-MB-468 cells, which have low endogenous CA levels (5–10% cells, data not shown), was tested. Interestingly, Centrinone B treatment of MDA-MB-468 cells did not significantly reduce migration or invasion (a reduction of 3% and 16%, respectively; Fig. S1D). This suggests that in cancer cells with high CA, the increased migration and invasion is dependent on increased CA signalling. However, in cancer cells with low CA, the increased migration and invasion is via signalling pathways that are independent of CA.

### CA induced migration and invasion involves activation of Rap1

It has been reported that activation of Rap1 (from inactive GDP-bound to active GTP-bound) can cause increased metastasis in cancer cells, playing a key role in regulating cell–ECM contacts (Sit and Manser, 2011). An activated Rap1 pulldown assay revealed that +CA MCF10A increased Rap1 activation (Fig. 2A).

Supporting this, CA increased cell–ECM attachment (in comparison to untreated cells, Fig. 2B). Demonstrating the importance of Rap1 activation in CA-induced ECM remodelling, treatment with a Rap1 inhibitor (GGTI-298) significantly (*P*≤0.01) decreased this CA-induced cell–ECM attachment (Fig. 2B). Also, GGTI-298 treatment significantly decreased both the CA-induced cell invasion and migration (Fig. 2C,D; Transwell assays). Furthermore, in the chick xenograft model, MCF10A PLK4 +CA cells treated with Rap1 inhibitor prior to engraftment showed a significant (*P*≤0.001) decrease in the CA-induced bidirectional chicken–graft focal reaction *in vivo* (Fig. 2E,F).

### CA induced disruption of epithelial barrier integrity

**Fig. 3.**
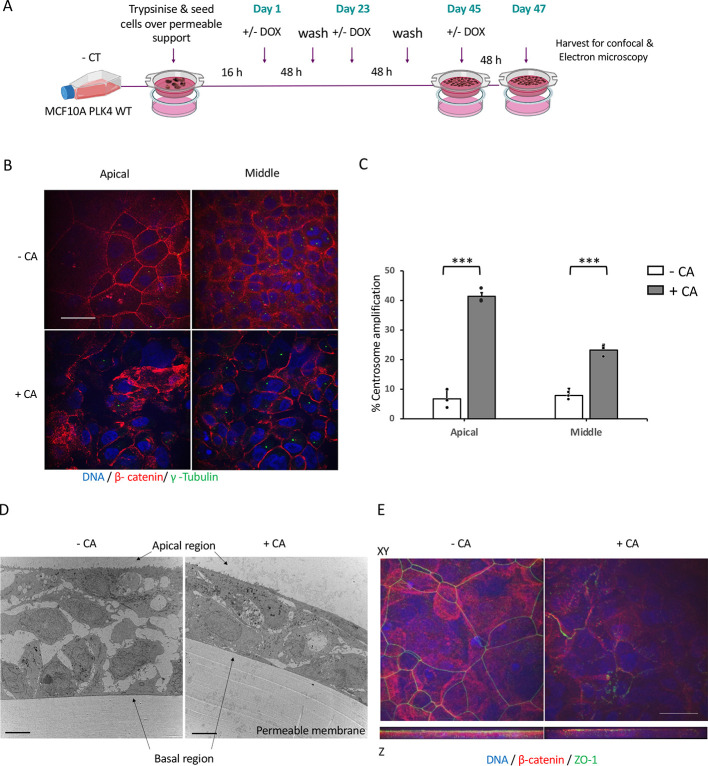
**Long-term MCF10A PLK4 wild-type cell culture Transwell system.** (A) Experimental layout of long-term Transwell membrane cell culture system. (B) Representative immunofluorescence images from four experiments of MCF10A PLK4 wild-type cells cultured for 47 days. CA was induced by treating at the specified intervals with 2 µg/ml Dox. The cells were fixed and permeabilised in 3.7% PFA and methanol/EGTA. Centrosomes were labelled for γ-tubulin (green) and AJs with β-catenin (red), and DNA counterstained with DAPI. Disrupted and mislocalised β-catenin are seen in +CA cells compared to –CA cells showing well-formed β-catenin at the apical region. Scale bar: 20 μm. (C) The bar graph shows a significant increase in percentage CA within the apical and middle layers of the multi-layered long-term culture. Bar graphs represent mean±s.e.m. from three biological repeats. ***P≤0.001 (unpaired two-tailed Student's *t*-test). (D) TEM images of sagittal view of the multi-layered cell structure with altered intercellular space in response to CA induction. Images representative of four independent experiments. Scale bars: 4 µm. (E) *XY* and *Z*-stack projections showing disruption of the TJ protein ZO-1 (green) and the AJ protein β-catenin (red) at the apical side in +CA conditions. Localisation of both ZO-1 and β-catenin was found to be primarily within the apical region rather than the middle or basal regions in long-term culture system. Scale bar: 20 μm.

Cell adhesiveness is dysregulated in tumours, resulting in defective and altered histological structure promoting cancer invasion and metastasis (Hirohashi and Kanai, 2003). The effects of CA on epithelial barrier integrity and apical junction complexes are unknown. We explored the effects of CA on cell–cell adhesion and cell–ECM adhesion. Under standard cell culture conditions, MCF10A cells form poor tight junctions (TJs) (Fogg et al., 2005; Underwood et al., 2006). Therefore, to study the effect of CA on apical junctional complexes (AJCs), an established long-term MCF10A cell culture assay, in which robust TJs are formed, was used (Fig. 3A) (Marshall et al., 2009). Long-term CA-induced cultures showed a significant increase (*P*≤0.001) in +CA MCF10A cells at the apical (42%) and middle (25%) regions of the multi-layered, apico-basally polarised cell culture system (Fig. 3B,C). CA induced disruption of β-catenin staining from well-ordered cortical rings to a disordered incomplete ring staining with focal condensations (Fig. 3B). Furthermore, a sagittal transmission electron microscopy (TEM) overview revealed CA induction induced clear morphological changes to the multi-layered polarised cell culture (Fig. 3D). CA induced spatial alterations to the adherens junction (AJ) protein β-catenin, showing a loss of basal β-catenin (in the apical lining cells of the cell layer), and reduction in apical TJ marker ZO-1 (also known as TJP1) (Fig. 3E).

### CA disrupts apical junctional complexes

**Fig. 4.**
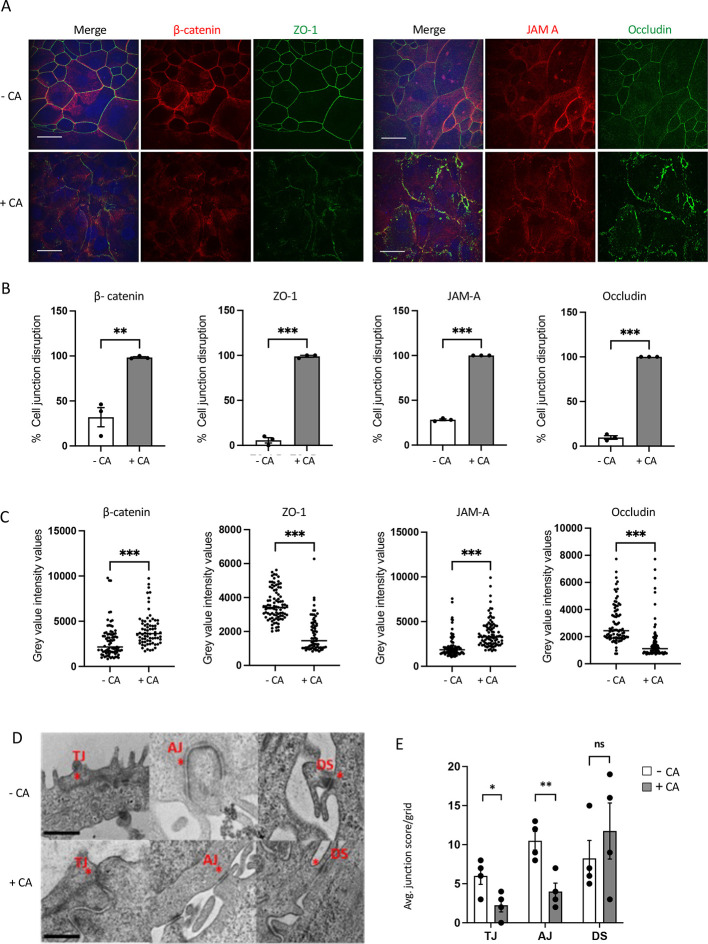
**CA disrupts apical junctional complexes in MCF10A PLK4 cells.** (A) Representative confocal images of cells co-stained for (left panels) the AJ protein β-catenin (red) and TJ protein ZO-1 (green), and (right panels) TJ proteins JAM-A (red), occludin (green) showing localisation and intactness of epithelial cell junctions in +CA and –CA MCF10A PLK4 cells. The cells were fixed in 3.7% PFA and permeabilised in 0.5% Triton X-100. Images are representative of three biological repeats. Scale bars: 20 μm. (B,C) Graphs represent mean±s.e.m. percentage cell junction staining disruption in 10 fields of view/slide (B) and average grey value intensity values over 10 fields of view (C). CA significantly disrupted cell junctions and altered the protein localisation. **P≤0.01, ***P<0.001 (paired two-tailed Student's *t*-test). (D) Representative TEM images of +CA and –CA TJs, AJs and DSs indicated by asterisk (*) taken at 20,000×, HV=100.0 kV. Scale bars: 600 nm. (E) Bar graph represents the cell junction quantification showing a significant downregulation of TJs and AJs (in apical region) and no significant difference in desmosomes due to CA. Bars represent mean±s.e.m. junction number from four independent experiments (10 images per experiment). *P<0.05, **P≤0.01; ns, not significant (unpaired two-tailed Student's *t*-test).

Correct localisation and orientation of cell junction proteins are required for maintaining epithelial cell polarity and epithelial barrier integrity, including the TJ proteins JAM-A (also known as F11R), occludin and ZO-1, and the AJ protein β-catenin. In addition to ZO-1 and β-catenin, CA disrupted the TJ family proteins occludin and JAM-A (Fig. 4A; Fig. S4A,B). Quantification of cell junction disruption revealed CA induced a significant increase in overall epithelial cell junction disruption (Fig. 4B; *P*≤0.01–*P*<0.001). Quantifying the effects of CA on epithelial barrier intensity and protein distribution revealed that there was a significant increase in (*P*<0.001) β-catenin and JAM-A intensity, and a significant decrease (*P*≤0.001) in ZO-1 and occludin intensity (Fig. 4C). Detailed analysis of the increased grey value intensity values induced by CA for β-catenin and JAM-A indicated a change to pockets of brighter, more punctate localisation of the protein, whereas ZO-1 and occludin showed an overall reduction in intensity of staining induced by CA. Graphs represent mean±s.e.m. from three independent experiments. Confirming these results obtained were specifically due to CA, and not off-target effects of PLK4 overexpression or DOX treatment, PLK4^1-608^ overexpression did not cause significant cell junction disruption or protein mislocalisation (Fig. S4C).

Using TEM analysis, TJs are observed as electron-dense apical membrane contact points or ‘kissing points’ where adjacent plasma membranes fuse and form a seal on the intercellular space (Farquhar and Palade, 1963). The less-electron dense AJs are located immediately below the TJs, also connecting to the actin cytoskeleton, whereas underlying desmosomes (DSs) connecting to microfilaments appear as electron rich surrounded by a fuzzy area. Ultra-structural investigation of CA effects on cell junction formation, revealed that DSs were the major intercellular junctions bridging inner cells. TJ/AJs were mainly found towards apical region whereas DSs appeared as small intracellular plaques abundant towards the interior and basal layers (Fig. 4D).

**Fig. 5.**
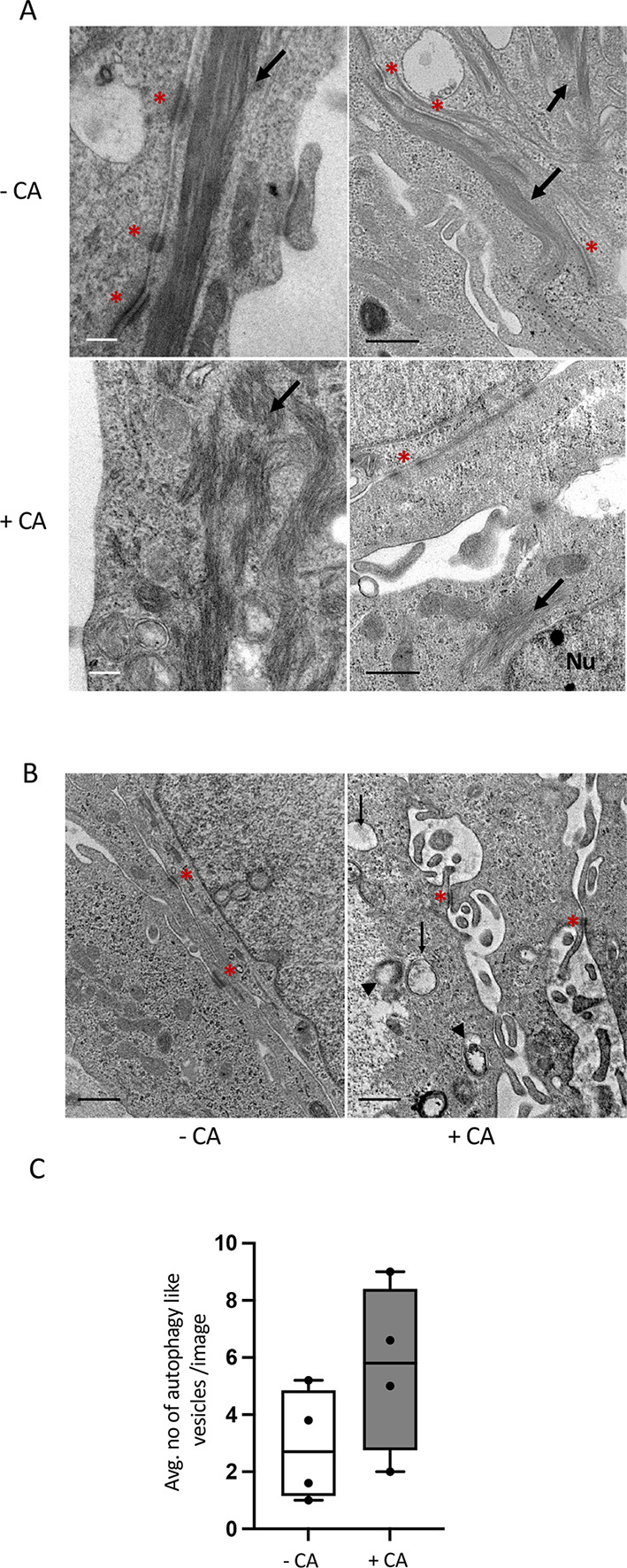
**CA alters cytoskeletal structure and increases phagophore and autophagosome-like structures.** (A) Representative images from three experiments of altered microfilament structures in long-term +CA MCF10A cultures. –CA cells show normal elongated filaments (indicated by arrows) and +CA show severely truncated, undulating filaments (indicated by arrows). Cell junctions (AJ and DS) are indicated by an asterisk (*), Nu, nucleus. Images were captured at 20,000×, HV=100.0 kV. Scale bars: 200 nm (white); 600 nm (black). (B) Representative images from three experiments of phagophore (arrows) and autophagosome-like (double membrane structure, arrowheads) structures in +CA cells found proximal to deformed cell junction complexes. DSs are indicated by asterisk (*). HV=100.0 kV. Scale bar: 500 nm. (C) Box plot of the number of phagophore/autophagosomes structures observed −CA and +CA in 15 fields of view. The box represents the 25–75th percentiles, and the median is indicated. The whiskers show the data spread from three independent biological repeats.

When quantifying the effects of CA on cell junctions, we found that +CA MCF10A had significantly reduced TJs and AJs (*P*≤0.05–*P*≤0.01) at the apical side cell–cell interface (Fig. 4E). We noted a definite but non-significant increase in DS numbers in inner cells of +CA MCF10A grids, suggesting a possible role for desmosomes in cell–cell contact in response to CA disrupted TJ/AJ formation. Furthermore, CA induction induced long bundles of cytoskeletal microfilaments, which were closely associated with intermediate cell junctions (DS), to become acutely truncated and structurally different (undulating) (Fig. 5A). Additionally, +CA MCF10A consistently displayed increased autophagosome or phagophore-like structures near deformed cell junctions (Fig. 5B,C). Intriguingly, these findings support work by Denu et al., demonstrating that CA disrupts autophagy trafficking by modulating the MT network and increases the accumulation of autophagosomes in MCF10A cells (Denu et al., 2020).

### CA disrupts epithelial cell–cell adhesion in tumorigenic breast cancer cells

**Fig. 6.**
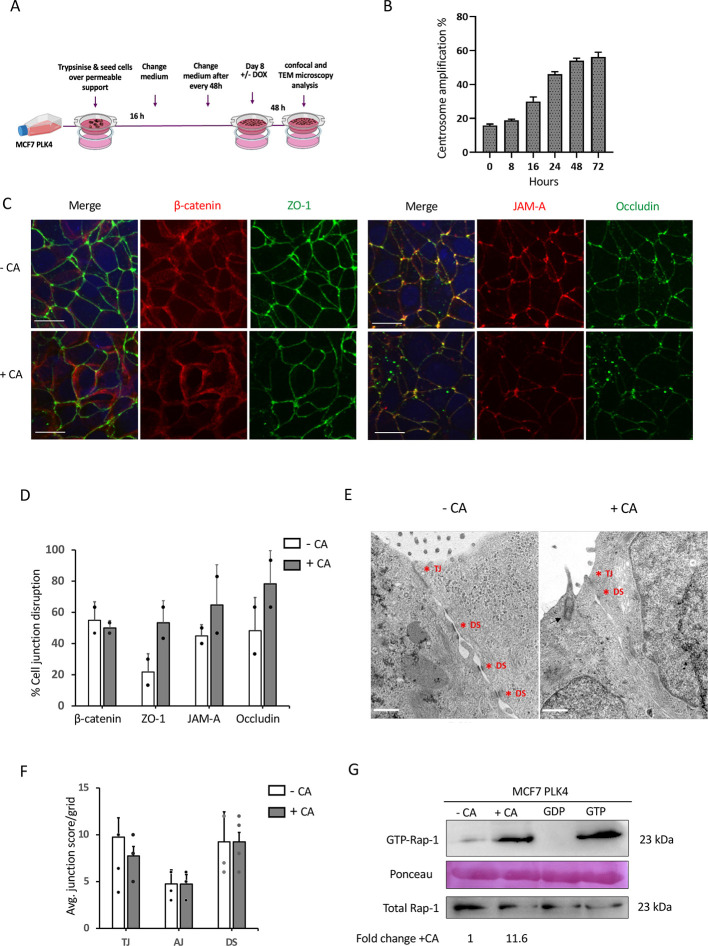
**CA disrupts epithelial cell–cell adhesion and increases active Rap1 levels in tumorigenic breast cancer cells.** (A) Experimental layout of a long-term MCF7 PLK4 cell culture system. (B) Bar graph shows percentage of +CA MCF7 PLK4 cells at each time point post-Dox (2 μg/ml). Cells were fixed with methanol/5 mM EGTA solution and stained for centrosomes (γ-tubulin, green) and MTs (α-tubulin, red), and counterstained with DAPI (DNA, blue). Bars represent mean±s.e.m., *n*=3; ≥200 cells/time point. (C) Representative images of +CA and –CA MCF7 cells co-stained for the AJ protein β-catenin (red), the TJ protein ZO-1 (green) (top panels), and the TJ proteins JAM-A (red) and occludin (green) (bottom panels). Scale bars: 20 μm. (D) Bar graph representing mean±s.e.m. the percentage cell junction staining disruption in 10 fields of view/slide. (E) Representative TEM images of MCF7 PLK4 cells showing cell junctions in +CA and –CA cells (indicated by asterisk). Cells were imaged at a magnification of 20,000×, HV=100.0 kV. Scale bars: 600 nm. (F) Bar graph represents cell junction quantification for +CA and –CA, with a clear decrease in TJ formation in +CA MCF7 PLK4 cells. Bars represent mean±s.e.m. cell junction number from four independent experiments. No significance was found (two-way ANOVA with Bonferroni post-test). (G) Western blot of Rap1 pulldown assay in MCF7 PLK4 cells showing –CA cells had increased GTP-bound Rap1. Negative (GDP) and positive (GTPγS) controls on untreated cell lysates were included. Ponseau stain is included as a loading control for the pulldown. Total Rap-1 in 60 µg of total cell lysates is shown in the bottom panel. The image shown is representative of *n*=3 independent repeats and the fold-change +CA represents the quantification of ±CA bands by densitometry.

To investigate the effects of CA on tumorigenic cells, we chose the luminal A breast cancer cell line MCF7, which assembles (partial) tight junctions under standard long-term culture conditions (Zangari et al., 2014). We used the same CA induction system of PLK4 overexpression in MCF7 PLK4 (Arnandis et al., 2018). CA was induced in a long-term culture system (Fig. 6A) in MCF7 PLK4 cells (+CA MCF7, Fig. 6B). In response to CA, quantification of epithelial TJ markers ZO-1, JAM-A and occludin revealed a clear, but non-significant disruption and mislocalisation (Fig. 6C,D). Also, diffusion of β-catenin away from the cortical membrane was observed in +CA MCF7 cells (Fig. 6C). Ultrastructural analysis on +CA MCF7 revealed that although CA had no effect on AJ or DS formation, there was a declining trend in TJ formation (Fig. 6E,F). Finally, as in MCF10A, CA increased Rap1 activation in MCF7 PLK4 cells (Fig. 6G).

### Profiling early-stage CA dysregulation of cell–cell junction and cell–ECM attachment proteins and genes

**Fig. 7.**
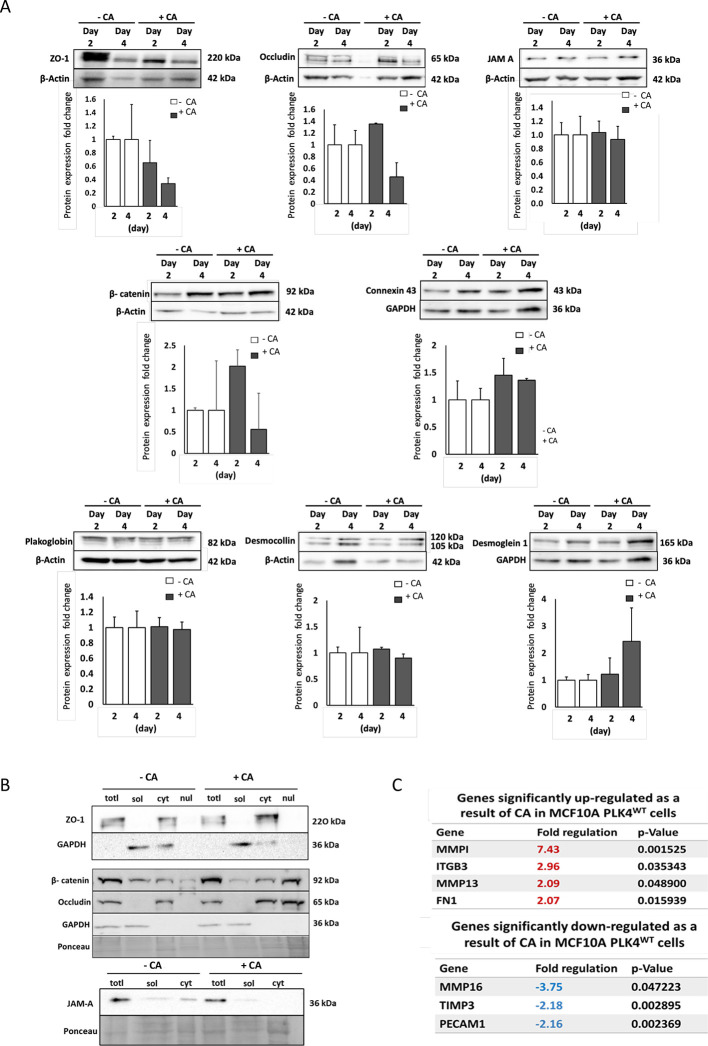
**CA dysregulation of cell–cell junction and cell–ECM attachment proteins and genes.** (A) Western blots on total protein extracts showing fold-change in expression above β-actin or GAPDH controls (mean±s.e.m., *n*=3). (B) Western blot of subcellular fractionation (*n*=3). totl, total; sol, cytosolic; cyt, cytoskeletal; nul, nuclear. CA caused altered recovery of TJ, ZO-1, occludin and JAM-A in cytoskeletal fractions and redistribution of β-catenin and occludin to the nuclear fractions. (C) In RT-PCR analysis of +CA and –CA MCF10A cells, CA significantly upregulated MMP1, MMP13, ITGB3, FN1 and downregulated MMP16, TIMP3 and PECAM1 gene expression. The table shows final data from Qiagen with the CT cut-off value as 35. The *P*-values were calculated by Student's *t*-test of the replicate 2^–ΔCT^ values for each gene in the control group and treatment groups (*n*=3 independent experiments).

To map the early stages of CA induction on mechanisms of cell attachment, cell junction formation and cell adhesion, cell junction total protein expression was evaluated at 48 and 96 h post-CA induction. A 5-fold reduction in total TJ ZO-1, and a 1.4-fold reduction in total TJ occludin was observed in +CA cells (Fig. 7A, upper panels). A ∼2-fold increase in total AJ β-catenin and a 1.5-fold upregulation of gap junction connexin 43 (Fig. 7A, middle panels) was seen +CA. DS desmoglein 1 displayed a 2-fold increase whereas DS desmocollin 2 and 3 were unaffected +CA. Plakoglobin (also known as γ-catenin), an adaptor protein found in both DS and AJ was unaffected (Fig. 7A, lower panels). Analysis of subcellular fractions showed that CA induction was associated with redistribution of occludin and β-catenin from the cytoskeletal to the nuclear fractions, with reduced levels of JAM-A in cytoskeletal fraction (Fig. 7B). Although ZO-1 levels increased in the cytoskeletal fraction, confocal and TEM analysis demonstrate that this is an abnormal, punctate localisation, which does not form a robust cell junction.

To further profile dysregulation in response to CA, transcriptional analysis of key cell adhesion and cell–ECM gene expression was performed (Fig. 7C). CA induction significantly upregulated (*P*=0.035) the expression of the cell–matrix adhesion molecule expression integrin β3 (ITGB3), its binding partner and ECM component fibronectin (FN1) (*P*=0.016). The MMP ECM-degrading enzymes were likewise significantly upregulated [MMP1 (*P*=0.001) and MMP13 (*P*=0.049)]. MMP16 levels were significantly downregulated (*P*=0.047) as were those of the MMP inhibitor (TIMP3) (*P*=0.003) and PECAM1 (*P*=0.002).

## DISCUSSION

CA is a known hallmark of cancer that is detected in a range of human solid and haematological malignancies, and is particularly associated with increased tumour aggressiveness, poor survival and metastatic cancer subtypes (Chan, 2011; Denu et al., 2016). This work supports mounting evidence that CA induction alone can induce tumorigenic transformation of normal cells (Rhys and Godinho, 2017). Here, we showed that CA promotes cell migration and invasiveness *in vitro*, independently of the mechanism of induction (PLK4 overexpression or CDK1 inhibition). This work quantifies the dissemination of +CA pro-tumorigenic cells out of the epithelial layer via a combination of cell autonomous and non-cell-autonomous effects of CA previously described by others (Arnandis et al., 2018; Godinho et al., 2014). This was supported by *in vivo* validation (using the chick embryo xenograft model; Harper et al., 2021), which demonstrated the earliest possible observable effects of CA induction, with cell invasion into the chicken mesoderm, hyperplasia of the chorionic epithelium at the interface with the Matrigel–cell graft, heterophil infiltrates (heterophils are analogous to mammalian neutrophils) and dilated lymphatics. Additional secondary tumour-associated characteristics of CA induction found *in vivo* included haemorrhaging, central necrosis and calcification. This clearly demonstrates that induction of CA alone, in a control non-tumorigenic background, is sufficient to trigger extensive pro-tumorigenic characteristics within days. These findings further inform the role of CA in tumorigenesis observed using mouse models (Coelho et al., 2015; Levine et al., 2017; Sercin et al., 2016).

Signalling pathways involving small GTPases, Rho and Ras, frequently shown to be dysregulated in tumours, have previously been proposed to mediate CA-induced cellular effects. The Rho subfamily consisting of RhoA, Rac1 and Cdc42 is well known for regulating cytoskeleton and actin dynamics, and Rac1 has been associated with lamellipodia formation during cell migration (Sit and Manser, 2011). Increased MT nucleation by supernumerary centrosomes upon CA induction leads to increased Rac1 activity, which promotes actin polymerisation disrupting cell adhesion and promoting cell invasion (Godinho et al., 2014). In addition, the small GTPase Ras-associated protein 1 (Rap1) is highly expressed in cancer cells, stimulating integrin-mediated cell–ECM attachment, MMP expression involved in ECM remodelling and cytoskeletal modulation, paving the way for cancer cell invasion and migration (Zhang et al., 2017). We found that CA induction increases active GTP-bound Rap1, and that CA-dependent cell–ECM attachment and invasion is highly dependent on this Rap1-mediated signalling. *In vivo*, Rap-1 inhibition blocked key tumorigenic phenotypes displayed by centrosome amplified cells (invasion, hyperplasia and dilation of lymphatics and blood vessels). Therefore, in addition to Rac1, the Rap1 GTPase plays a key role in the signalling pathways mediating CA induction of an invasive phenotype in control breast epithelial cells, and this points to Rap1 inhibition as a potential new therapeutic option for many CA tumours. Rap1 acts as a tumour promoter mediating metastasis in a range of cancer contexts, including breast, prostate, ovarian, oesophageal and pancreatic cancers (reviewed in Looi et al., 2020) and this work informs the mechanism by which Rap1 works.

Our work shows that CA induction in an untransformed epithelial background is sufficient to trigger migration and invasion *in vitro* and *in vivo*, in conjunction with elevated cell–ECM attachment. Previous work has shown that tumorigenesis is associated with dramatic alterations in integrin expression profiles, with these adhesion molecules facilitating cell movement by forming focal adhesions between cancer cells and the ECM (reviewed in Hamidi and Ivaska, 2018). In breast cancer, integrin β3 overexpression (bound to subunit αv to form αvβ3 integrin) is associated with high metastatic potential, especially in promoting bone metastasis (Carter et al., 2015; Havaki et al., 2007; Kovacheva et al., 2021). Integrin β3 overexpression in breast epithelia paves the way for invading cells by inducing secretion of MMP1 and MMP2 with resultant matrix degradation. Fibronectin binding to integrin β heterodimeric receptors stimulates the production of MMP1, inducing an MMP1-dependent invasive phenotype in breast cancer cells (Jia et al., 2004). Our data show CA-induced upregulation of gene expression of *ITGB3* alongside that of its ECM-binding partner fibronectin, and dysregulation of the balance of ECM-degrading enzymes MMPs (MMP1, MMP13 and MMP16) and tissue inhibitors of MMPs (TIMP3). These effects of CA support clinical studies reporting that high fibronectin expression in metastatic breast cancer is associated with high MMP expression (Das et al., 2008; Fernandez-Garcia et al., 2014).

The formation of apico-basal polarised epithelia is closely coupled to cell–cell adhesion events at AJCs, consisting of TJs and the sub-adjacent AJ, helping regulate normal cell polarity and movement and inhibiting abnormal migration and metastasis (Wang and Margolis, 2007). TJs act as a semi-permeable adhesive barrier regulating traffic through the paracellular space (gate function) while physically segregating lipids between apical and basolateral compartments. AJs contribute to cell–cell adhesion via an adhesive belt lying basally to TJs. The cytoplasmic domains of the proteins that comprise AJC junctions are tethered to the cytoskeleton via various adaptor proteins integrating signals to regulate cell polarity and movement. We observed that CA disrupted and mislocalised TJ and AJ components in MCF10A and MCF7 cell lines. Lack of statistical significance for these effects in the tumorigenic MCF7 background might be due to compensatory mechanisms activated by existing endogenous levels of CA harboured by these cells. The subcellular mislocalisation of JAM-A, occludin and β-catenin away from the cytoskeleton, as well as overloading of ZO-1 protein at the cytoskeleton in +CA cell populations, is a likely underlying cause of disrupted of cell–cell adhesion. It is likely that excessive centrosome numbers impact on the cytoskeleton protein dynamics via elevated MT nucleation followed by actin polymerisation (Godinho et al., 2014). Intriguingly, MT polymerisation inhibitors (MTIs) can re-establish functional AJCs in cell–cell adhesion-defective carcinoma cells via mechanosensitive recruitment of junction molecules (Ito et al., 2017). In that case, GTPase RhoA was found to be upregulated by MTIs, restoring AJCs by causing actomyosin contraction at the apical cortex and tension-dependent junction reassembly. The possibility that the opposite effect might be happening in our +CA cells with elevated Rap1-GTP regulating RhoA activity remains to be explored.

The treatment of transformed cancer cell lines with the PLK4 inhibitor Centrinone B to decrease inherent CA and reverse CA-induced phenotypes has previously been demonstrated (Denu et al., 2020, 2018; Wong et al., 2015). In the current study, differential effects of Centrinone B on invasive capabilities of the cells correlated with endogenous CA levels, where the confirmed contribution of CA to migration and invasion of triple negative MDA-MB-231 breast epithelial cells (high CA) was not recapitulated in MDA-MB-468 (low CA). This indicates that across cancer cell subtypes, there are varying contributions from CA and other cellular mechanisms driving invasive capacity.

In conclusion, we found that CA induces tumorigenic changes in breast epithelial contexts *in vitro* and *in vivo*, promoting the transition to an invasive phenotype via disruption of cell-cell contacts and release of stromal proteases to facilitate destruction of the basement membrane and increased motility of invasive cells through the ECM. The *in vivo* finding that CA is sufficient to confer such advanced tumorigenic changes within days, identifies CA as an early driver alteration in cancer and a central targetable mechanism.

## MATERIALS AND METHODS

### Cell culture

Wild-type MCF10A (ATCC) and MCF10A PLK4, MCF10A^1-608^ doxycycline-inducible PLK4 cell lines (a gift from Susana Godinho, Barts Institute, London, UK; Godinho et al., 2014) were used to model CA in an untransformed breast cell context following 48 h doxycycline treatment at 2 mg/ml. MCF10A cells were cultured in Dulbecco's modified Eagles' medium/F-12 medium supplemented with 5% horse serum (Sigma 14M175/ H1270), 0.5 μg/ml hydrocortisone (Sigma H0888), 20 ng/ml human epidermal growth factor (hEGF) (Sigma E9644), 10 μg/ml insulin (Sigma I9278) 1% penicillin-streptomycin (Sigma P4333). The breast cancer cell lines MDA-MB-231, MDA-MB-468 (ATCC) and doxycycline-inducible MCF7 PLK4 cells (a gift from Susana Godinho; Arnandis et al., 2018) were cultured in RPMI-1640 (Sigma R8758) supplemented with 10% fetal bovine serum (Sigma F7524) and 1% penicillin-streptomycin (Sigma P4333). For CA induction, cells were treated with 2 µg/ml doxycycline hyclate (Sigma D9891) or 10 µM of the CDK1 inhibitor RO3306 (Sigma SML0569). 150 nM Centrinone B (Biotechne, R&D systems, Tocris, 5690) treatment for 16 h inhibited CA. Rap1 was inhibited with 10 µM GGTI-298 (Biotechne Tocris 2430) for 3 h.

### Immunofluorescence microscopy

Cells were seeded on poly-D-lysine (Sigma P7280) coated coverslips and fixed in chilled methanol/EGTA (5 mM; Sigma E4378) at −20°C. Cells were incubated with primary antibodies against centrosome (γ-tubulin; Sigma T3559) and MT (α-tubulin; Sigma B512) markers and stained with secondary antibodies (Alexa Fluor^®^ 594, Invitrogen A-11005, Alexa Fluor^®^ 488, anti-rabbit-IgG; Invitrogen A-11008). Coverslips were mounted in Vectashield with DAPI (Vector Laboratories H-1200) and stored at 4°C. Imaging was performed on the OLYMPUS BX61 fluorescent microscope driven by Olympus cellSens imaging software and *Z*-stacks (0.4 µm) captured. Centrosome number was quantified in ≥200 cells per slide categorising normal as 1–2 centrosomes/cell or centrosome amplification as ≥3 centrosomes.

### Cell invasion and migration assays

CA was induced for 36 h with 2 µg/ml DOX in MCF10A PLK4 then cells serum-deprived (1% horse serum and no hEGF) overnight (12 h). Cells were seeded on Transwell inserts (8-μm pore, Corning 353097) – coated with Matrigel (Corning™ 356231) for invasion assay; uncoated for migration assay. To create a gradient, complete media with 6% horse serum and 20 ng/ml hEGF was added to the lower chamber. For negative gradient controls, serum-deprived medium (1% horse serum and no hEGF) was added to the upper and lower chambers. Cells were incubated for 12 h and 24 h to measure cell migration and invasion, respectively. Cells on the underside of the filter post-incubation were stained and quantified using Crystal Violet (Sigma HT901-8FOZ). Brightfield images from five fields of view per insert were captured on the OLYMPUS CKX31, under 20× magnification. Migrated and invaded cells were quantified using ImageJ software wand tool (Schneider et al., 2012).

### Chicken embryo xenograft model

The *in vivo* model we use is the chicken egg chorioallantoic membrane (CAM) assay; all relevant animal legislation was followed (European law Directive 2010/63/EU). Accordingly, *in ovo* experiments we have used here do not require any special additional allowance as long as the embryos are euthanized before two-thirds of gestation. Hatching occurs at day 21; therefore we culled at day 13.

Fertilised chicken eggs (Breed: Ross 308, three eggs per treatment) were incubated at 37°C and 65% humidity. On embryonic development (ED) day 3, a window was opened in the eggshell under a heat lamp and 2.5 ml albumen aspirated off using a syringe. The eggs were re-sealed with transparent adhesive film (Opsite Flexifix™, Smith-Nephew). On ED day 8, MCF10A PLK4 +CA or −CA and with or without 10 µM GGTI-298 in 1× PBS–Matrigel (Corning: 354248; 50:50 ratio) was implanted onto the chicken membrane within a sterile silica ring. On ED day 13, the embryos were killed, and the cell–Matrigel graft with attached CAM harvested. Gross images were captured using a stereo microscope (Leica S6E), and the tissue fixed and paraffin-embedded in the Thermo Scientific™ Excelsior Tissue Processor unit. 5 µm tissue sections were prepared using a Microtome (Microm HM325, Thermo Fisher Scientific), mounted and stained with haematoxylin and eosin stain (H&E) and imaged as *Z*-stacks by brightfield microscopy with a 40× objective on the Olympus VS120 Virtual Slide Scanner Microscope. The VS120 scans the entire slide or selected area, equipped with autofocus capabilities to capture multiple images with the upmost positional accuracy, compensating for variations in slide thickness. In cases where the scanned area is larger than a single camera's field of view, the advanced software algorithms ‘stitch’ the images together to form a reliable, seamless high-resolution composite panorama image. Areas of interest can be manually identified for high resolution imaging, omitting those areas that have either no tissue or are of less relevance to speed up the scanning process.

### Immunohistostaining of xenograft tissue

The anti-cytokeratin 8 monoclonal antibody (Cytokeratin CAM 5.2 BDBiosciences-345779) was used to label MCF10A cell invasion into the chicken membrane. Slides were deparaffinised, blocked and incubated with anti-Cyt8 antibody (1:2 dilution), HRP-conjugated secondary antibody followed by 3,3′-diaminobenzidine (DAB) staining using the ImmPRESS™ Excel Amplified HRP peroxidase Polymer Staining Kit (Vector Laboratories MP-7602-15), according to the manufacturer's protocol. All images were captured as Z-stacks of brightfield microscopy with a 40× objective on the Olympus VS120 Virtual Slide Scanner Microscope.

### Long-term Transwell cell culture

The process for long-term cell culture in MCF10A PLK4^1-608^ cells was adapted from Marshall et al. (2009). MCF10A PLK4^1-608^ cells cultured in complete DMEM/F12 medium in the absence of cholera toxin (CT) were seeded onto the Transwell inserts (1.2×10^5^ cells, pore size 0.4 µm). The cells were maintained by replacing the medium in both chambers every 48 h. Previous studies have reported that CA prevalence is higher in *in vivo* tumours than in *in vitro* cells due to intratumoural hypoxia, and that cells with high CA levels *in vitro* are eliminated over time (Mittal et al., 2017). To maintain levels of CA, MCF10A were pulsed with Dox (2 µg/ml) at regular intervals (Day 1, 23, 45) of the long-term culture (Fig. 3A).

MCF7 PLK4 cells were analysed in a 10-day Transwell cell culture system (Fig. 6A) adapted from that shown in Sheller et al. (2017).

Transwell inserts were sliced into quarters with a sharp scalpel blade and mounted onto Superfrost slides. Cells were fixed with 3.7% paraformaldehyde and permeabilised with 0.5% Triton X-100 in PBS and hybridised with primary antibodies against the cell junction proteins JAM-A (sc-53623, 1:500 dilution), β-catenin (sc-7963, 1:1000 dilution), occludin (Invitrogen 71-1500, 1:1000) or ZO-1 (Invitrogen 61-7300, 1:1000), and secondary antibodies (Alexa Fluor^®^ 594 anti-mouse-IgG; Invitrogen A-11005, Alexa Fluor^®^ 488 anti-rabbit-IgG; Invitrogen A-11008). Filters were mounted with Vectashield plus DAPI, and immunofluorescence images captured using an Andor revolution spinning disk confocal microscope under 60× magnification. The cell junction integrity on the apical side was quantified using binary scoring method, 1=intact cell junction and 0=disrupted cell junction. Cell junction protein localisation was quantified using the value intensity (maximum intensity projection of stacks converted to greyscale) of cell junctions marked by β-catenin, ZO-1, JAM-A and occludin, at the apical region for ten fields of view, using the ImageJ FIJI Broadly Applicable Routines (BAR) plugins.

### TEM

Long-term cultures of MCF10A PLK4^1-608^ and MCF7 PLK4 seeded on filter inserts were fixed in 2% glutaraldehyde (Sigma G5882), 2% paraformaldehyde (Thermo Fisher Scientific 10131580) in 0.1 M sodium cacodylate pH 7.2 (Sigma C0250). Filters were washed in 0.2 M sodium cacodylate buffer, incubated in 1% osmium tetroxide in 0.1 M sodium cacodylate buffer (pH 7.2). Filters were dehydrated through a graded series of ethanol concentrations and infiltrated with resin–acetone mixtures overnight (50:50 mixture, 75:25 mixture and 100% resin). Ultra-thin (70–90 nm) sections were cut in a sagittal plane using a diamond knife and mounted onto 3 mm copper grids. Grids were counterstained with UA Zero non-radioactive EM Stain (Agar Scientific AGR1000) and imaged on a Hitachi 7500 TEM microscope under 20,000× magnification. Cell junctions were quantified in ten images per grid (four grids per treatment in four independent experiments) and a binary score of 1 and 0 assigned to presence or absence of each cell junction, respectively.

### Immunoblotting

Whole-cell lysates were prepared as previously described (Gao et al., 2014). A subcellular protein extraction was performed as described in Choi et al. (2014). Briefly, cells were lysed in 50 mM PIPES, 50 mM NaCl, 5% glycerol, 0.1% NP-40, 0.1% Triton X-100 and 0.1% Tween 20 with a protease inhibitor cocktail (Sigma P8340). The supernatant (cytoplasmic fraction) was retained, and cells rinsed in 5 ml of 50 mM Tris-HCl (pH 7.4) and nuclease buffer containing 10 U/ml Benzonase^®^ nuclease (Sigma E1014), 10 mM MgCl_2_ and 2 mM CaCl_2_ in 50 mM Tris-HCl buffer, pH 7.5. The supernatant was retained (nuclear fraction), and cytoskeletal proteins collected by solubilising the pellet in 0.1% SDS.

The protein concentration was determined by Pierce™ Bicinchoninic acid (BCA) Protein Assay according to the manufacturer's instructions. Western blotting was carried out with standard protocols. Primary antibodies used were against JAM-A (sc-53623, 1:500), β-catenin (sc-7963, 1:1000), occludin (Invitrogen 71-1500, 1:1000), ZO-1 (Invitrogen 61-7300, 1:1000), plakoglobin (BD 61025, 1:1000), desmoglein-1 (BD 610273, 1:500), Desmocollin 2/3 (Zymed 32-6200, 1:500), Connexin 43 (Proteintech 15386-1-AP, 1:500), anti-actin (Sigma-Aldrich A2066, 1:5000) and anti-GAPDH (Sigma-Aldrich G9545, 1:5000). Secondary antibodies used were HRP-conjugated anti-mouse and rabbit - Jackson ImmunoResearch Laboratories Inc.

### Rap-1 activation pulldown assay

MCF10A or MCF7 cells were seeded in 150 mm dishes and CA was induced with 2 μg/ml Dox for 48 h. The assay was performed using the Rap1 Activation Assay Kit (17-321 Sigma-Aldrich) according to the manufacturer's instructions.

### RNA preparation and real-time quantitative PCR

The Qiagen RT2 Profiler PCR Array for 84 Cell Adhesion and Extracellular Matrix molecules (PAHS-013Z) was used and the PCR component mix for 96 well plate prepared according to manufacturer's instructions. The experiment was performed as per the Profiler array format C protocol on a StepOnePlus™ Real-Time PCR System (Applied Biosystems). Data analysis was carried out using the Qiagen GeneGlobe RT-qPCR Data Analysis web tool.

### Cell adhesion assay

The cell adhesion assay was performed as previously described in Chen (2012). Briefly, at 48 h of CA induction using 2 µg/ml Dox and overnight serum starvation, cells were transferred to type I collagen (Corning™ 354231) (positive control) or 1% BSA (negative control)-coated 96 well plates and incubated for 20 min. After a series of washing steps, the cells bound to the substrate were quantified by Crystal Violet staining and the ImageJ software wand tool (Schneider et al., 2012).

### Statistical analyses

All statistical comparisons were performed with GraphPad^®^ Prism 9 (www.graphpad.com). Depending on the number of factors, multiple-group comparisons were made using either a one- or two-way analysis of variance (ANOVA) followed by the Bonferroni test to confirm statistical differences between groups. Paired sample analysis was performed using a two-sided unpaired or paired Student's *t*-test. Statistical significance (*P*-value) was assigned for values <0.05.

## Supplementary Material

10.1242/joces.261150_sup1 Supplementary information
