# Supplementary material for: Centrosome amplification promotes cell invasion via cell–cell contact disruption and Rap-1 activation
Source: J Cell Sci. 2023 Nov 1;136(21):jcs261150. doi: 10.1242/jcs.261150 (PMC10629695; doi:10.1242/jcs.261150)
Supplement: Supplementary information [file joces-136-261150-s1.pdf]

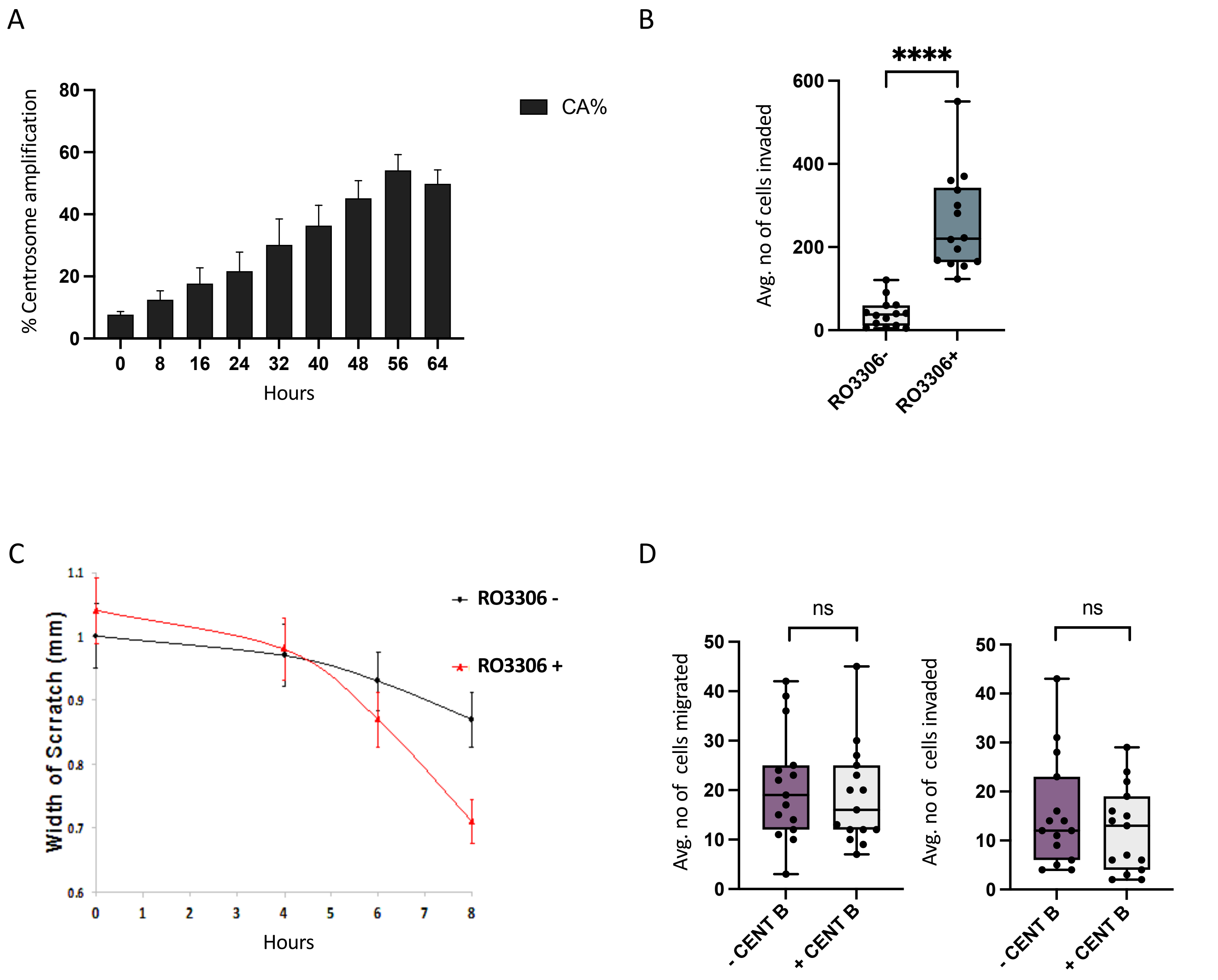

Fig. S1. CA induced by CDK1 inhibition increases *in vitro* migration and invasion and blocking CA does not effect metastatic characteristics of MDA-MB-468. (A) Bar graph representing % cells +CA in WT MCF10A post CDK1 inhibitor RO3306 (10 µM) treatment. Bars represent mean ± SEM from 3 independent experiments, ≥200 cells/time point. (B) CA increases cellular invasion and (C) migration in WT MCF10A. (D) Blocking CA using Centrinone B in TNBC MDA-MB-468 cells (with low endogenous CA) did not significantly effect migration and invasion.

Journal of Cell Science • Supplementary information

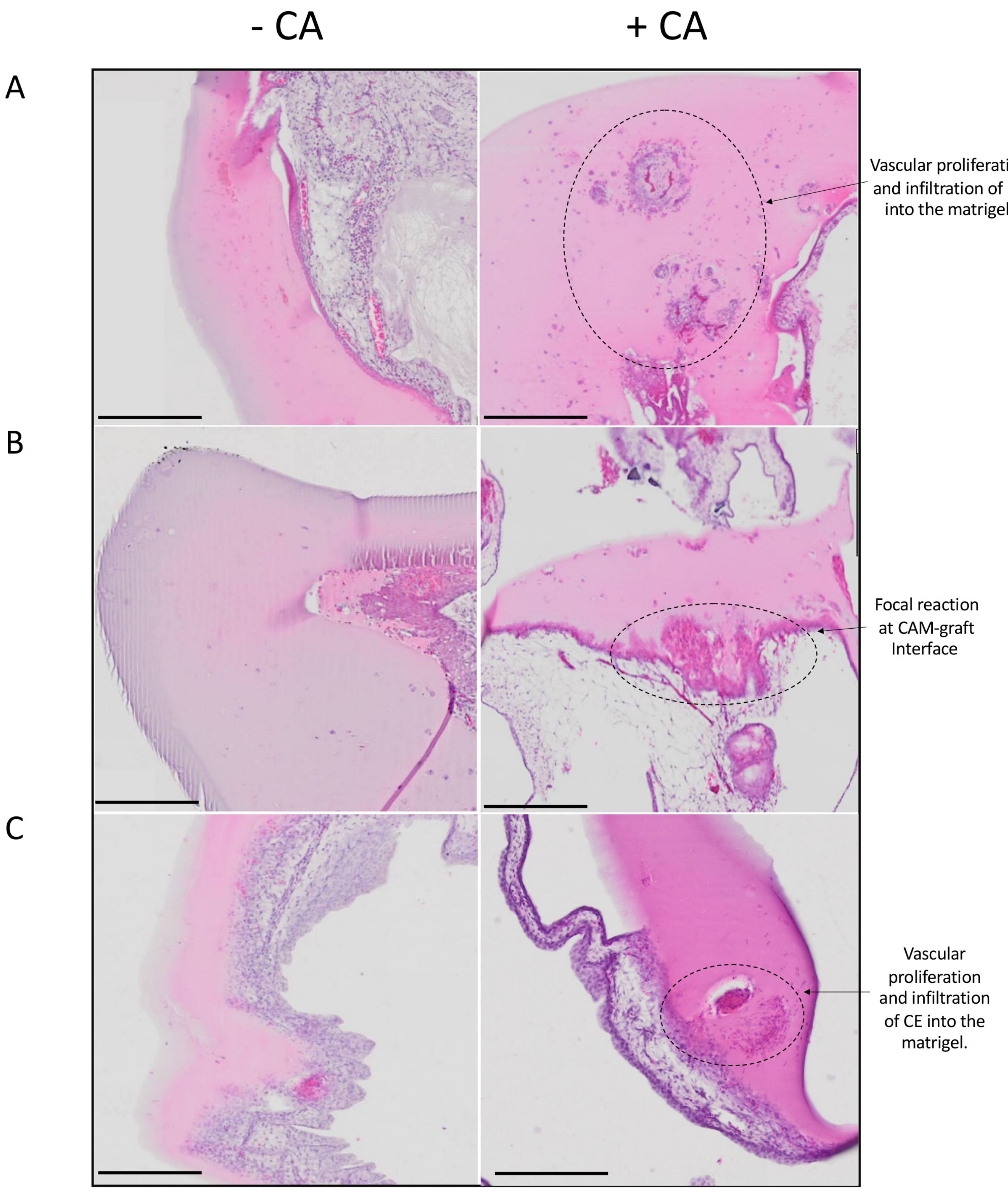

Fig. S2. Representative images of chicken xenograft model ± CA. (A-C) Further representative images of the excised ±CA MCF10A-matrigel grafts onto the chicken chorioallantoic membrane (n=4). +CA grafts show marked reactions of the chick chorioallantoic membrane to the matrigel-cell graft with hyperplasia and inflammatory infiltrate**.** In (A) and (C), the +CA sample shows vascular proliferation and infiltration of the CE into the matrigel layer. Zero or a mild reaction was observed in the -CA grafts.

Journal of Cell Science • Supplementary information

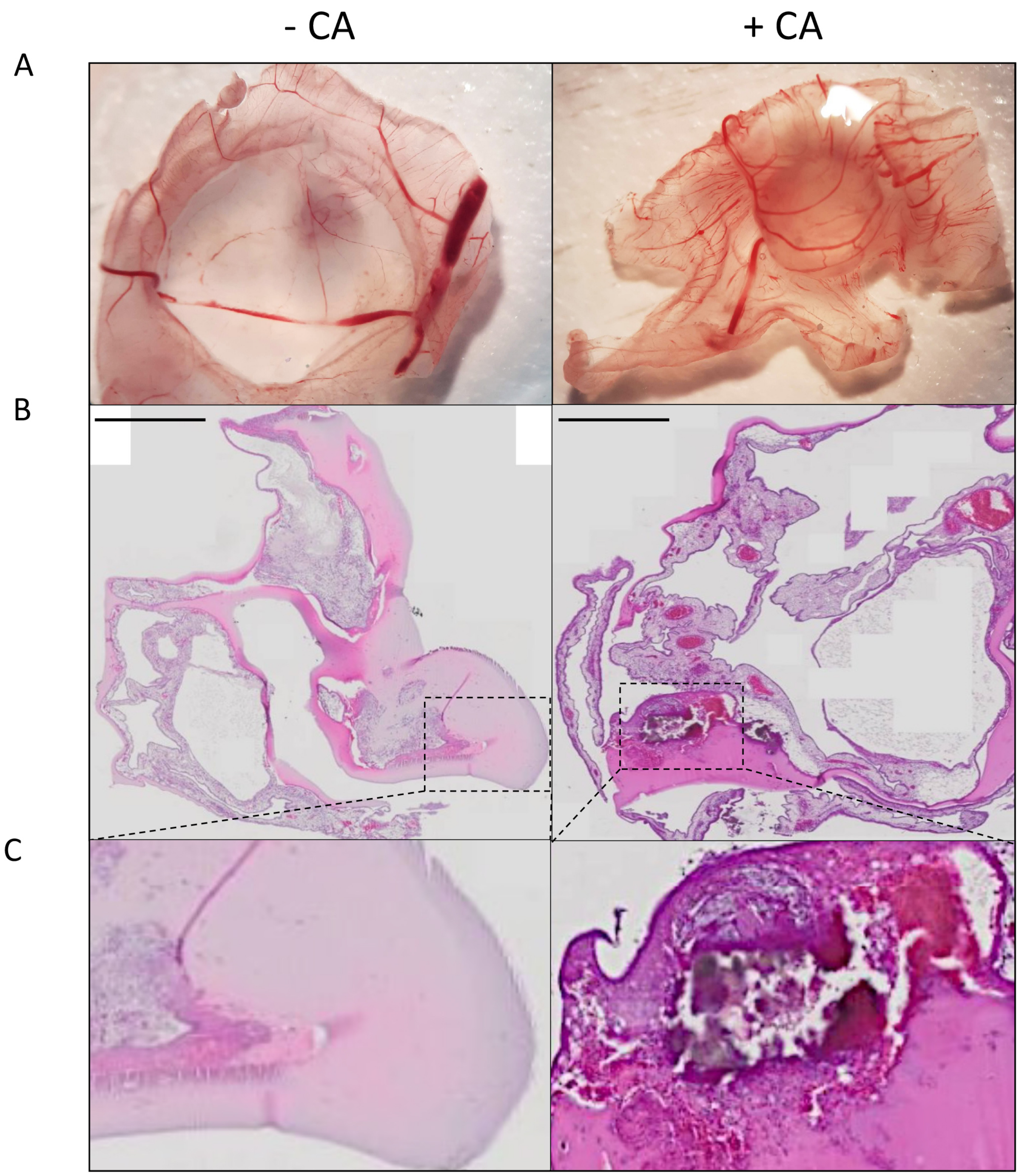

Fig. S3. CA induces early secondary tumour characteristics in chicken xenograft model (A) Gross images of excised ±CA MCF10A PLK4/matrigel graft captured on a stereo microscope (Leica S6E). (B) +CA cell engraftment causes multifocal moderate to marked reaction of the chick chorioallantoic membrane to the Matrigel in contrast to a zero to mild reaction seen in the -CA graft. (C) The +CA sections (enlarged box) show increased epithelial hyperplasia, inflammatory infiltrate and secondary tumour characteristics including vascular proliferation, haemorrhaging with accompanying central necrosis. Images in (B) and (C) are composites of `stitched' 40X images created by the Olympus VS120 slide scanner (40X) with advanced software algorithms. Areas of interest are manually identified (focussed area in C) for high-resolution imaging, omitting those areas which have either no tissue or are of less relevance.

Journal of Cell Science • Supplementary information

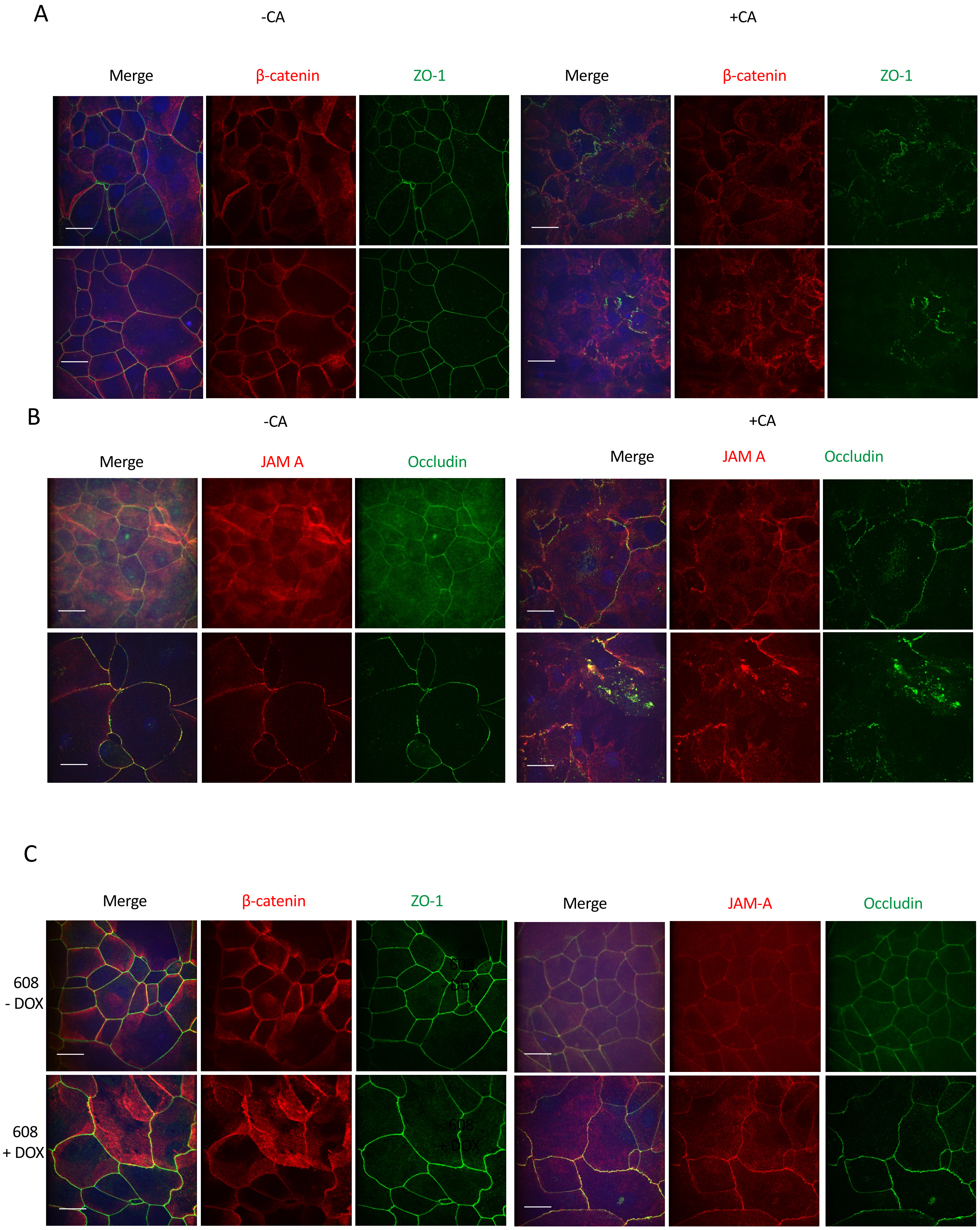

Fig. S4. Doxycycline treatment and overexpression of truncated PLK4$^{1\text{-}608}$ variant has no effect on localisation of tight junction and adherens junction proteins. Additional representative images long-term cell cultures. (A-B) Immunofluorescent co-staining of MCF10A PLK4 ±CA shows combinations of AJ protein β-catenin (red), TJ protein ZO-1 (green), and TJ proteins JAM-A (red), occludin (green). Images are representative of 4 independent biological repeats. Scale bar 20µm. (C) Representative images of long-term cell cultures of negative control MCF10A$^{\mathbf{1\text{-}608}}$ ± Dox co-stained for AJ protein β-catenin (red), TJ protein ZO-1 (green) and TJ proteins JAM-A (red), occludin (green) (n=2). Scale bar 20µm. Doxycycline treatment and overexpression of PLK4$^{1\text{-}608}$ did not disrupt apical junction complex protein localisation.

Journal of Cell Science • Supplementary information
